# Is Type 2 Diabetes Really Resolved after Laparoscopic Sleeve Gastrectomy? Glucose Variability Studied by Continuous Glucose Monitoring

**DOI:** 10.1155/2015/674268

**Published:** 2015-04-14

**Authors:** D. Capoccia, F. Coccia, A. Guida, M. Rizzello, F. De Angelis, G. Silecchia, F. Leonetti

**Affiliations:** ^1^Department of Experimental Medicine, Division of Diabetes and Metabolic Diseases, Policlinico Umberto I, 00161 Rome, Italy; ^2^Department of Medical Surgical Sciences and Biotechnology, Division of General Surgery, ICOT, Sapienza University of Rome, 04100 Latina, Italy

## Abstract

The study was carried out on type 2 diabetic obese patients who underwent laparoscopic sleeve gastrectomy (LSG). Patients underwent regular glycemic controls throughout 3 years and all patients were defined cured from diabetes according to conventional criteria defined as normalization of fasting glucose levels and glycated hemoglobin in absence of antidiabetic therapy. After 3 years of follow-up, Continuous Glucose Monitoring (CGM) was performed in each patient to better clarify the remission of diabetes. In this study, we found that the diabetes resolution after LSG occurred in 40% of patients; in the other 60%, even if they showed a normal fasting glycemia and A1c, patients spent a lot of time in hyperglycemia. During the oral glucose tolerance test (OGTT), we found that 2 h postload glucose determinations revealed overt diabetes only in a small group of patients and might be insufficient to exclude the diagnosis of diabetes in the other patients who spent a lot of time in hyperglycemia, even if they showed a normal glycemia (<140 mg/dL) at 120 minutes OGTT. These interesting data could help clinicians to better individualize patients in which diabetes is not resolved and who could need more attention in order to prevent chronic complications of diabetes.

## 1. Introduction

The prevalence of type 2 diabetes mellitus (T2DM) is rapidly increasing worldwide in parallel with the current obesity epidemic. In 2010 the global prevalence of type 2 diabetes was estimated at 8.3% of the adult population and 23% of patients with morbid obesity have type 2 diabetes. These data are projected to increase and prevalence of diabetes is expected to reach 9.9% by 2030 [1]. Conventional medical treatment of type 2 diabetes may achieve only partial glycemic control and reduction of cardiovascular risk [2], especially if associated with obesity. As known, management of diabetes is particularly challenging in morbidly obese patients.

Although initially developed solely as a weight reduction therapy, bariatric surgery has been reported to cure and improve type 2 diabetes and to reduce rates of cardiovascular disease and death [3–6].

A meta-analysis of studies [7] on various bariatric procedures involving patients with type 2 diabetes showed an overall rate of remission of hyperglycemia of 78% among the various procedures. Remission occurred in approximately half of patients who underwent Laparoscopic Adjustable Gastric Banding (LAGB), 80% of those who underwent Roux-en-Y gastric bypass, and 95% of those who underwent biliopancreatic diversion (BPD).

Data regarding diabetes remission following Laparoscopic Sleeve Gastrectomy (LSG) are extremely variable amongst authors. In a meta-analysis [8] that analyzed 27 studies and 673 patients, diabetes remission was reported in 66.2% of cases and improvement was reported in 26.9% of cases. In our previous studies diabetes remitted in 80% of patients [9, 10], results that have been predominantly confirmed by a recently published meta-analysis by Yu et al. [11].

Busetto et al. showed a direct correlation between weight loss and the remission of diabetes following various surgical procedures [12].

Bariatric literature usually presents results in terms of rate of diabetes cure or remission, usually defined as the normalization of glucose levels and glycated hemoglobin (A1c) in the absence of active antidiabetic therapy [13]. In analogy to the oncologic literature, where cure is defined as complete remission of cancer of sufficient duration that the future risk of recurrence is felt to be very low, experts agreed that it may make sense, on a practical ground, to consider prolonged (arbitrarily 5 years) remission of diabetes essentially equivalent to cure. Experts agreed that it may make sense, on a practical ground, to consider prolonged (arbitrarily 5-year) remission of diabetes essentially equivalent to cure.

In this context, authors have defined the following conditions. “Partial remission” is A1c < 6.5% and fasting glucose between 100 and 125 mg/dL for a period of at least 1 year's duration in the absence of active pharmacologic therapy.

“Complete remission” is a return to “normal” values of glucose metabolism (A1c < 6%, fasting glucose < 100 mg/dL) for at least 1 year's duration. “Prolonged remission” is complete remission that lasts for more than 5 years [13].

However, the remission or reoccurrence of diabetes after surgery is difficult to establish. Oral glucose tolerance tests (OGTT) are difficult to interpret both because of the modified glucose kinetics after surgery and because of the lack of reference data which define a “normal” OGTT after bariatric surgery.

Another question that arises from bariatric literature is the correct diagnosis of diabetes, both before and after surgery. Diagnosis of diabetes should be always performed according to ADA (American Diabetes Association) guidelines [14] which, besides other criteria, take into account a detection of random glycemia over 200 mg/dL during the day. Bariatric surgeons usually assess the presence of type 2 diabetes mellitus before surgery on the basis of the existing oral treatment regimen of patients (e.g., metformin treatment); following surgery they often define diabetes remission on the basis of fasting glycemia values, without a specific consideration to postprandial glycemic peaks.

It is now widely known that acute fluctuations of glucose around a mean value over a daily period of intermittent hyperglycemia may play an important role in the development of cardiovascular disease in type 2 diabetic patients [15, 16].

Fasting plasma glucose is usually normalized in postbariatric patients, while little is known about their glucose profiles throughout the day, especially immediately after meals, and about how this variability can affect the development of future micro- and macrovascular complications.

In literature, only few cases undergoing gastric bypass have been assessed through Continuous Glucose Monitoring (CGM). These cases show early hyperglycemic peaks after either a meal test or a glucose load, followed by a rapid fall to very low glycemic levels, with postprandial symptoms as seen in dumping syndrome. However, these authors have focused their attention only on the use of CGM in relation to hypoglycemia resulting from the operation [17, 18]. Regarding LSG, there are few recent data about diabetes remission investigated by CGM [19].

The aim of this study was to evaluate glucose variability assessed by CGM in obese type 2 diabetic patients that underwent LSG, who were defined as cured of diabetic disease according to international criteria, which consist in fasting glycemia < 100 mg/dL and A1c < 6% in absence of hypoglycemic therapy.

## 2. Subjects and Methods

### 2.1. Study Design

#### 2.1.1. Recruitment

A total of 20 morbidly obese patients were enrolled 3 years following LSG to perform CGM to evaluate glucose plasma variability and OGTT for glucose tolerance evaluation.

Patients were recruited at the Centre of Diabetes and Metabolic Diseases of Sapienza University in Rome. Exclusion criteria were overt diabetes or use of plasma glucose-lowering drugs.

All the patients were diabetics before surgery with T2DM diagnosed according to American Diabetes Association guidelines [14].

#### 2.1.2. Clinical Outcomes

The primary clinical outcome was evaluation of real diabetes remission after LSG by assessing glucose variability by CGM and glucose tolerance by OGTT.

The follow-up lasted at least 42 months consisting in clinical examinations with anthropometric measurements and routine laboratory tests with glucose metabolism assessment every 3 months. Diabetes remission after LSG was then defined as a fasting glucose level of less than 100 mg/dL and an A1c level of less than 6% in absence of treatment.

The results were then related with retrospectively collected data, referred to each patient before surgery in order to look for prognostic factors of the diabetes resolution obtained by LSG, including anthropometric parameters, duration of diabetes, age, BMI, insulin use, and fasting C-peptide as marker of insulin secreting ability.

#### 2.1.3. Variables

The following parameters were recorded at baseline and three years after surgery:

- Age, gender, body mass index (BMI), and systolic and diastolic pressure.
- Ongoing drug treatments.
- Fasting blood levels of glucose, A1c, total and HDL cholesterol, and triglycerides.

#### 2.1.4. Measurements

Standing height, weight, and waist circumference were measured with subjects wearing light clothing. Obesity was estimated by means of BMI [weight (kg) divided by height × height (m^2^)].

Blood pressure (mmHg) was measured after five minutes of rest with the patient sitting in the upright position. Three measurements were obtained, and the average of the second and third ones was recorded and used in the analysis. Patients underwent fasting blood sampling to assess blood glucose, A1c, total cholesterol, high-density lipoprotein cholesterol (HDL), and triglycerides, by standard laboratory methods carried out by our center.

#### 2.1.5. Surgical Procedure

LSG was performed according to National Institutes of Health criteria for bariatric surgery indications [20]. The surgical operation was applied as the previously described technique [21] in which five trocars are needed. The division of the gastric greater curvature vascular supply, starting at 6 cm from the pylorus and proceeding upwards until the angle of His, is carried out with radiofrequency (Harmonic Scalpel, Ethicon Endo­Surgery) or with Caiman 5 mm vessel sealing device (Aesculap). The gastric resection was performed using a linear stapler (ECHELON FLEX ENDOPATH STAPLER, Ethicon Endo­Surgery), with two sequential green load firings for the antrum, followed by three or four sequential gold loads for the remaining gastric corpus and fundus. The stapler is applied alongside a 42 Fr calibrating bougie strictly positioned against the lesser curve, to obtain an 80–100 mL gastric volume. The last shot of stapling was fired at least 1 cm distal to the esophagogastric junction. Reinforcement of the staple lines was performed with bioabsorbable strips (GORE SEAMGUARD Bioabsorbable Staple Line Reinforcement). Intraoperative methylene blue test was routinely performed at the end of the procedure to check for gastric leak and to evaluate the volume and the shape of the sleeve and a drain was routinely placed. The resected stomach was extracted in a retrieval bag.

#### 2.1.6. CGM

The CGM sensor (Medtronic iPro 2 digital recorder, MMT-7741) was attached to the abdomen of the patients by a serter and was held in place for 6 days. The first CGMS calibration was performed 1 h after initialization using blood from a finger prick. By using a glucometer, patients were asked to record three daily glycemic measurements in a diary that were necessary to obtain correct calibration of the recorder. The CGM profiles of the patients were then analyzed with software that enables automatic extraction of the necessary data and subsequent generation of graphs and the area under the curves. On day 6, the subjects returned to the hospital in the morning in fasting state for an oral glucose tolerance test (OGTT); measurement of plasma glucose at 0′-30′-60′-90′-120′ was performed during the CGM; and the CGM was stopped 2 hours after glucose load. In each patient the total AUC for glycemia during OGTT was calculated. During the CGM, the percentage of time conventionally spent in physiological range (70–140 mg/dL), under the physiological range (<70 mg/dL), and in the hyperglycemic range (>140 mg/dL) was assessed. AUC (area under the curve) above the limit of 140 mg/dL and AUC below the limit of 70 mg/dL were calculated during the CGM. In addition, standard deviation, as index for glycemic variability, glycemic peaks, and nadir during daily life, was evaluated.

#### 2.1.7. Ethics

The study was reviewed and approved by the institutional human ethics committee in accordance with national guidelines and the provisions of the Helsinki Declaration, as revised in 2000. All patients provided written informed consent to participate in the study, and additional written informed consent was obtained before any surgical procedure.

#### 2.1.8. Statistics

Clinical characteristics of the study participants were reported as mean and SD for continuous variables and frequencies and percentages for categorical variables. Statistical analyses were performed using SPSS software version 15.0 (SPSS, Inc., Chicago, IL, USA). Statistical significance was set at *P* < 0.05.

## 3. Results

All patients included in the study had been defined as cured according to the international criteria for diabetes remission [13]. Anthropometric and biochemical parameters and diabetes duration and treatment, before and after LSG, are reported in Table 1.

The data obtained by CGM were analyzed and showed a very different pattern of glycemic curves, suggesting that we differentiate patients with hyperglycemic values (conventionally defined >140 mg/dL) from the others who did not spend time in hyperglycemia. Therefore, we divided subjects into two groups.

**Figure 1:**
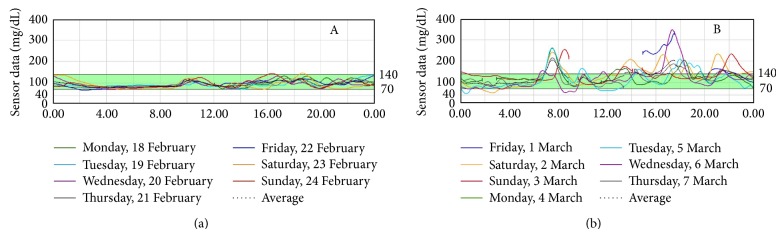
CGM profile in one patient of group A and in one patient of group B.

- Group A: patients who spent no time with glycemia over 140 mg/dL (example of CGM of one patient of this group is reported in Figure 1(a)).
- Group B: patients who spent part of their time in hyperglycemia, defined over 140 mg/dL (example of CGM of one patient of this group is reported in Figure 1(b)).

Patients of group A were defined as “cured” of diabetes and patients of group B were defined as “not cured.”

Glycemic peak and nadir during CGM are reported for both groups of patients in Table 2.

In patients of group A (8/20), 2 h OGTT showed physiologic glycemic peak at 30 minutes and at 120′ glycemia was <140 mg/dL. These patients were then considered “cured” of diabetes either by international criteria for diabetes remission for bariatric patients or by OGTT criteria, showing a normal glucose tolerance. In these subjects, during everyday life conditions, glycemia assessed by CGM never exceeded the 140 mg/dL levels.

In patients of group B (12/20), 2 h OGTT showed a delayed glycemic peak not earlier than 60 minutes and sometimes delayed at 90 minutes; 8 out of 12 patients of this group achieved normal levels at 120′ OGTT (<140 mg/dL), whilst the other patients remained higher than 200 mg/dL as usually occurs in diabetic patients. These patients spent from 13% to 45% of time with glycemia higher than 140 mg/dL during the CGM. Therefore, even if these patients showed a normal fasting glucose and normal A1c values, we decided to define them as “not cured” from diabetes.

**Figure 2:**
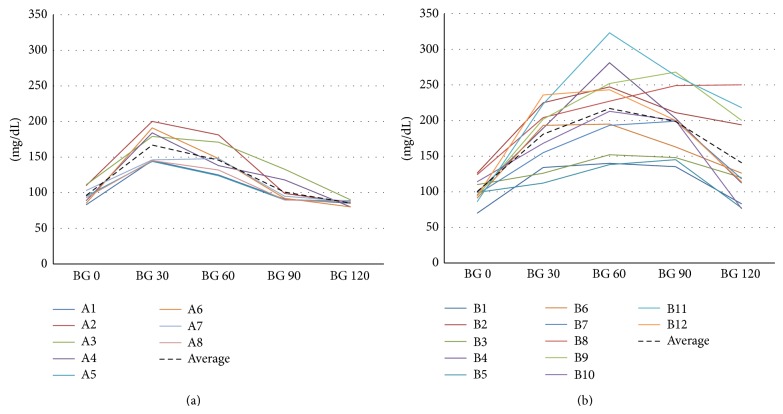
Glycemia during 2 h OGTT in each patient of group A (a) and group B (b).

2 h OGTT glycemic levels in each single patient of both groups are reported in Figure 2.

International criteria to exclude diabetes (fasting glycemia, A1c) and data obtained from OGTT and CGM in groups A and B are reported in Table 2.

The 2 h OGTT glycemia AUC in the two intervals of 0–120 minutes and 0–90 minutes has been calculated and added in Table 2 in groups A and B. The interval of 0–90 minutes has been investigated in consideration of the rapid gastric emptying due to LSG.

Retrospective assessment of anthropometric parameters, insulin use, A1c, fasting plasma C-peptide as marker of insulin secreting ability before surgery, and duration of diabetes was then compared in groups A and B as reported in Table 3.

## 4. Discussion

Since the pioneering reports of the last decade of the twentieth century [22, 23], the efficacy of bariatric procedures in improving and even normalizing glucose levels in obese patients with type 2 diabetes has been confirmed by a large number of observational studies [7].

However, while fasting plasma glucose concentrations are usually normalized in these patients, little is known about their glucose profiles throughout the day and particularly after meals.

In literature, there are only a few reports that adopt CGM following bariatric surgery, mainly aiming to assess hypoglycemia due to dumping syndrome after gastric bypass [17, 18].

This study adds something new to the knowledge of glucose variability and tolerance using the CGM and OGTT in patients that underwent LSG. It aims to assess glycemia throughout the day, as a means of investigating the real remission of diabetes, considering that fasting glucose and A1c might be insufficient to exclude the persistence or relapse of diabetes after surgery.

According to international guidelines on the definition of glycemic outcomes after bariatric surgery, all patients of this study had been defined as “cured” from diabetes 3 years after LSG.

However, we found that an unexpected 60% of these patients experienced significant hyperglycemia revealed by CGM during their everyday life, and in some cases overt diabetes was seen with glycemia > 200 mg/dL at 120 minutes of OGTT.

The results obtained by OGTT and CGM allowed confirming that patients appear really cured of diabetes only in group A (40%). In fact according to CGM these patients spent no time with glycemia over 140 mg/dL, even after meals. Surprisingly, a number of patients (group B) showed variable time with glycemia over 140 during CGM, often associated with very high glycemic postprandial levels, lasting approximately 40% of daily time. They also had a delayed glycemic peak at 60 minutes after oral glucose load which remained high until 90 minutes.

In literature, data are not available to clarify the correct interpretation of OGTT after bariatric surgery; the lack of this information might be one of the reasons the test is not included in the criteria of diabetes remission in bariatric operated patients. In our opinion, it is not reasonable to apply the standard OGTT criteria in operated subjects because the surgical procedure causes modified glucose kinetics due to an accelerated transit of food in the stomach [24].

In “cured” patients, who did not spend any time in hyperglycemia during CGM, glycemic OGTT peak occurred at the first measurement at 30 minutes, while at 90 minutes these patients showed glycemic values similar to basal values. On the contrary, in “not cured” patients, who spent a long time in hyperglycemia during CGM, the glycemic peak during OGTT occurred at 60–90 minutes with very high values. This effect demonstrates impaired insulin secretion in these patients as suggested by preoperative C-peptide levels. The massive secretory response of beta-cells to these very high and durable glycemic levels is able to rapidly reduce the glycemic levels to values of less than 140 mg/dL, suggesting normal glucose tolerance. In fact, these patients might be considered at high risk of diabetes recurrence. Therefore, at 2 h after glucose load, plasma glucose determinations may be misleading, and assessment of glycemia at thirty-minute intervals throughout the test is important.

Four diabetic patients of group B were defined as “cured” according to fasting glycemia and A1c before performing OGTT and CGM, but, in fact, elevated OGTT glycemic values diagnostic for overt diabetes (glycemia > 200 mg/dL at 120 minutes) were found. Furthermore, these patients spent about 40% of time during CGM with glycemia higher than 140 mg/dL and occasionally even higher than 200 mg/dL.

Recent studies using CGM showed significant fluctuations in blood glucose values even in patients with excellent A1c plasma levels. These data suggest that glucose variability may have a predictive role for the development of the complications of diabetes [25, 26]. Moreover Kilpatrick et al. [27] analyzed the Diabetes Control and Complications Trial (DCCT) patient cohort and observed that blood glucose instability is not a predictor of microvascular complications (mainly retinopathy) [28], but, on the other hand, mean daily glucose values, as well as the pre- and postprandial glucose ones, are predictors of cardiovascular disease [29].

Data are less consistent in T2DM. Several years ago, Muggeo et al. found that all-cause and cardiovascular mortality in elderly people with diabetes [30] were primarily associated with the variability/instability of glucose levels, rather than its absolute values.

The clinical use of CGM in this study allowed us to monitor the daily glycemic profile after LSG, which revealed unexpected hyperglycemia. By using CGM we found that only 40% of patients had complete hyperglycemia remission after LSG, although all patients had been considered “cured” according to existing standard definitions of glycemic outcomes after bariatric surgery. Our hypothesis is that A1c was not reliable in these cases, probably because of the short duration of hyperglycemic peaks that may have been too rapid for glycation of hemoglobin, even when glycemic peaks were very high.

Furthermore, we did not observe a balance between hyper- and hypoglycemia, as reported in gastric bypass surgery [17, 18] that could explain normal A1c values. In fact, in patients that underwent LSG, the percentage of time spent with glycemia less than 70 mg/dL was only 3.9% in all patients, exactly 5% in group A and 2.6% in group B.

In order to identify prognostic factors for complete diabetes remission, we compared the two groups prior to surgery by considering age, diabetes duration, beta-cell function, and insulin use.

There is generally agreement among the published studies that patients who have long-standing T2DM have a lower remission rate after bariatric surgery, probably due to their poor residual beta-cell function [31]. Other preoperative patient factors have been associated with the outcomes of diabetes remission, including age, fasting C-peptide concentration, BMI, glycemic control (A1c), and medications used to manage blood glucose, including oral hypoglycemic agents and insulin [32].

As described in a previous study [33], diabetes duration is the main prognostic factor for diabetes remission after LSG. In fact, we published the fact that patients with history of less than 10 years of diabetes were cured from diabetes, according to standard criteria for diabetes remission (e.g., fasting glucose (<100 mg/dL) and A1c (<6%)).

We observed that although all patients had a duration of diabetes of less than 10 years, patients of group A had a shorter diabetes duration (maximum 2 years) than patients of group B. Furthermore, patients with diabetes remission after LSG were younger, with higher fasting plasma levels of C-peptide, with higher BMI, and without insulin therapy before surgery.

The limits of this study were small sample size and small subgroups according to CGM results, and data was collected only after surgery. Further evaluations in a larger number of diabetics with morbid obesity, studied before and after the bariatric surgical operations, will be performed in order to give us more details on this topic.

## 5. Conclusions

Bariatric surgery significantly improves the prognosis of obese patients and improves the overall glycemic control in obese diabetics. We reported glucose variability in a real-life setting for the first time, assessed by CGM after LSG in patients defined as “cured” of diabetes according to international guidelines on remission of diabetes after bariatric procedures. The clinical relevance of this study consists in an insight into glycemia levels in carefully monitored diabetic patients that underwent LSG. We recommend 2 h OGTT, checking glycemia not only at 120 minutes, but also at every 30 minutes, in patients that are apparently cured of diabetes by standard criteria but that are still exposed to significant glucose variability which could cause future chronic complications of diabetic disease.

## Figures and Tables

**Table 1:** Characteristics of morbidly obese patients with T2DM before and after LSG at time of CGM.

	Before LSG	After LSG	*P*
Gender (F/M)	15/5	15/5	—
Age (years)	50.9 ± 8.2	53.1 ± 9.2	—
Weight (kg)	122.7 ± 32.1	78.8 ± 18.6	0.005
BMI (kg/m^2^)	44.3 ± 8.2	29.7 ± 6.1	0.003
Fasting glucose (mg/dL)	151.8 ± 37.4	83.5 ± 10.7	0.001
A1c (%)	7.4 ± 1.3	5.7 ± 0.6	0.035
Oral hypoglycemic agents/insulin	17/3	0/0	0.015
Diabetes duration (years)	5.3 ± 4.9	—	—

**Table 2:** Criteria for diabetes remission (fasting glycemia and A1c) and CGM and OGTT data in groups A and B.

	Group A	Group B	*P*
BG fasting (mg/dL)	79.7 ± 11.8	87.1 ± 8.5	n.s.
A1c (%)	5.5 ± 0.4	5.6 ± 0.5	n.s.
BG at 120′ OGTT (mg/dL)	87.3 ± 4.2	147.1 ± 59.4	0.04
0′–120′ glycemia AUC during OGTT (mg dL^−1^ min)	17902.5 ± 5552.1	19940.4 ± 5149.1	n.s.
0′–90′ glycemia AUC during OGTT (mg dL^−1^ min)	12345.6 ± 20008.3	16587.0 ± 3502.8	0.02
CGM time with glycemia >140 mg/dL (%)	0	21.4 ± 8.06	0.002
CGM AUC plasma glucose >140 mg/dL (mg dL^−1^ min)	0	8.0 ± 6.2	0.06
CGM time with glycemia <70 mg/dL (%)	5 ± 1	2.6 ± 0.8	n.s.
CGM AUC plasma glucose <70 mg/dL (mg dL^−1^ min)	0.7 ± 0.1	0.14 ± 0.05	n.s.
CGM glycemic peak (mg/dL)	156.2 ± 12.6	241.2 ± 70.5	0.03
CGM glycemic nadir (mg/dL)	52.2 ± 12.2	63.1 ± 11.1	n.s.
CGM variability (SD)	16.8 ± 5.24	30.0 ± 9.9	0.02

**Table 3:** Differences between group A and group B regarding age, BMI, insulin therapy, A1c, C-peptide, diabetes duration, and weight loss.

	Group A	Group B	*P*
Age (years)	48.4 ± 8.2	52.0 ± 8.5	0.02
Preoperative BMI (kg/m^2^)	45.1 ± 7.2	42.8 ± 7.4	0.04
Insulin use (number of patients)	0/8	3/12	—
A1c before surgery (%)	7.5 ± 1.0	7.4 ± 1.0	—
C-peptide before surgery (ng/mL)	5.2 ± 1.5	3 ± 0.5	0.02
Diabetes duration before surgery (years)	2.5 ± 2.3	6.5 ± 4.5	0.04
Total weight loss after surgery (%)	33.7 ± 13.9	31.6 ± 11.8	—
EWL after surgery (%)	63.8 ± 26.3	69.1 ± 16.8	—

## References

[1] International Diabetes Federation (2011). *IDF Diabetes Atlas*.

[2] Liebl A., Mata M., Eschwège E. (2002). Evaluation of risk factors for development of complications in Type II diabetes in Europe. *Diabetologia*.

[3] Sjöström L., Lindroos A.-K., Peltonen M. (2004). Lifestyle, diabetes, and cardiovascular risk factors 10 years after bariatric surgery. *The New England Journal of Medicine*.

[4] Sjöström L., Narbro K., Sjöström C. D. (2007). Effects of bariatric surgery on mortality in Swedish obese subjects. *The New England Journal of Medicine*.

[5] Sjöström L., Peltonen M., Jacobson P. (2012). Bariatric surgery and long-term cardiovascular events. *The Journal of the American Medical Association*.

[6] Mingrone G., Panunzi S., De Gaetano A. (2012). Bariatric surgery versus conventional medical therapy for type 2 diabetes. *The New England Journal of Medicine*.

[7] Buchwald H., Estok R., Fahrbach K. (2009). Weight and type 2 diabetes after bariatric surgery: systematic review and meta-analysis. *The American Journal of Medicine*.

[8] Gill R. S., Birch D. W., Shi X., Sharma A. M., Karmali S. (2010). Sleeve gastrectomy and type 2 diabetes mellitus: a systematic review. *Surgery for Obesity and Related Diseases*.

[9] Leonetti F., Capoccia D., Coccia F. (2012). Obesity, type 2 diabetes mellitus, and other comorbidities: a prospective cohort study of laparoscopic sleeve gastrectomy vs medical treatment. *Archives of Surgery*.

[10] Abbatini F., Capoccia D., Casella G., Coccia F., Leonetti F., Basso N. (2012). Type 2 diabetes in obese patients with body mass index of 30–35 kg/m^2^: sleeve gastrectomy versus medical treatment. *Surgery for Obesity and Related Diseases*.

[11] Yu J., Zhou X., Li L. (2015). The long-term effects of bariatric surgery for type 2 diabetes: systematic review and meta-analysis of randomized and non-randomized evidence. *Obesity Surgery*.

[12] Busetto L., Sbraccia P., Frittitta L., Pontiroli A. E. (2011). The growing role of bariatric surgery in the management of type 2 diabetes: evidences and open questions. *Obesity Surgery*.

[13] Brethauer S. A., Aminian A., Romero-Talamás H. (2013). Can diabetes be surgically cured? Long-term metabolic effects of bariatric surgery in obese patients with T2DM. *Annals of Surgery*.

[14] American Diabetes Association (2013). Standards of Medical Care in Diabetes-2013. *Diabetes Care*.

[15] Ma C.-M., Yin F.-Z., Wang R. (2011). Glycemic variability in abdominally obese men with normal glucose tolerance as assessed by continuous glucose monitoring system. *Obesity*.

[16] Wang C., Lv L., Yang Y. (2012). Glucose fluctuations in subjects with normal glucose tolerance, impaired glucose regulation and newly diagnosed type 2 diabetes mellitus. *Clinical Endocrinology*.

[17] Ritz P., Vaurs C., Bertrand M., Anduze Y., Guillaume E., Hanaire H. (2012). Usefulness of acarbose and dietary modifications to limit glycemic variability following Roux-en-Y gastric bypass as assessed by continuous glucose monitoring. *Diabetes Technology and Therapeutics*.

[18] Hanaire H., Bertrand M., Guerci B., Anduze Y., Guillaume E., Ritz P. (2011). High glycemic variability assessed by continuous glucose monitoring after surgical treatment of obesity by gastric bypass. *Diabetes Technology and Therapeutics*.

[19] Jiménez A., Ceriello A., Casamitjana R., Flores L., Viaplana-Masclans J., Vidal J. (2015). Remission of type 2 diabetes after Roux-en-Y gastric bypass or sleeve gastrectomy is associated with a distinct glycemic profile. *Annals of Surgery*.

[20] (1991). NIH consensus statement covers treatment of obesity. *The American Family Physician*.

[21] Basso N., Capoccia D., Rizzello M. (2011). First-phase insulin secretion, insulin sensitivity, ghrelin, GLP-1, and PYY changes 72 h after sleeve gastrectomy in obese diabetic patients: the gastric hypothesis. *Surgical Endoscopy*.

[22] Pories W. J., Swanson M. S., MacDonald K. G. (1995). Who would have thought it? An operation proves to be the most effective therapy for adult-onset diabetes mellitus. *Annals of Surgery*.

[23] MacDonald K. G., Long S. D., Swanson M. S. (1997). The gastric bypass operation reduces the progression and mortality of non-insulindependent diabetes mellitus. *Journal of Gastrointestinal Surgery*.

[24] Rodieux F., Giusti V., D'Alessio D. A., Suter M., Tappy L. (2008). Effects of gastric bypass and gastric banding on glucose kinetics and gut hormone release. *Obesity*.

[25] Frontoni S., di Bartolo P., Avogaro A., Bosi E., Paolisso G., Ceriello A. (2013). Glucose variability: an emerging target for the treatment of diabetes mellitus. *Diabetes Research and Clinical Practice*.

[26] Salardi S., Zucchini S., Santoni R. (2002). The glucose area under the profiles obtained with continuous glucose monitoring system relationships with HbA1c in pediatric type 1 diabetic patients. *Diabetes Care*.

[27] Kilpatrick E. S., Rigby A. S., Atkin S. L. (2006). The effect of glucose variability on the risk of microvascular complications in type 1 diabetes. *Diabetes Care*.

[28] Kilpatrick E. S., Rigby A. S., Atkin S. L. (2009). Effect of glucose variability on the long-term risk of microvascular complications in type 1 diabetes. *Diabetes Care*.

[29] Kilpatrick E. S., Rigby A. S., Atkin S. L. (2008). Mean blood glucose compared with HbA_1c_ in the prediction of cardiovascular disease in patients with type 1 diabetes. *Diabetologia*.

[30] Muggeo M., Verlato G., Bonora E., Zoppini G., Corbellini M., de Marco R. (1997). Long-term instability of fasting plasma glucose, a novel predictor of cardiovascular mortality in elderly patients with non-insulin-dependent diabetes mellitus: the verona diabetes study. *Circulation*.

[31] Lee W. J., Hur K. Y., Lakadawala M. (2013). Predicting success of metabolic surgery: age, body mass index, C-peptide, and duration score. *Surgery for Obesity and Related Diseases*.

[32] Dixon J. B., Chuang L.-M., Chong K. (2013). Predicting the glycemic response to gastric bypass surgery in patients with type 2 diabetes. *Diabetes Care*.

[33] Casella G., Abbatini F., Calì B., Capoccia D., Leonetti F., Basso N. (2011). Ten-year duration of type 2 diabetes as prognostic factor for remission after sleeve gastrectomy. *Surgery for Obesity and Related Diseases*.

